# The Role of Non-coding RNAs in the Pathogenesis of Glial Tumors

**DOI:** 10.32607/actanaturae.11270

**Published:** 2021

**Authors:** T. F. Kovalenko, T. D. Larionova, N. V. Antipova, M. I. Shakhparonov, M. S. Pavlyukov

**Affiliations:** Shemyakin-Ovchinnikov Institute of Bioorganic Chemistry Russian Academy of Sciences, Moscow, 117997 Russia

**Keywords:** glioma, glioblastoma, long noncoding RNAs, circRNAs, miRNAs, piRNAs, snRNAs, snoRNAs

## Abstract

Among the many malignant neoplasms, glioblastoma (GBM) leads to one of the worst prognosis for patients and has an almost 100% recurrence rate. The only chemotherapeutic drug that is widely used for treating glioblastoma is temozolomide, a DNA alkylating agent. Its impact, however, is only minor; it increases patients’ survival just by 12 to 14 months. Multiple highly selective compounds that affect specific proteins and have performed well in other types of cancer have proved ineffective against glioblastoma. Hence, there is an urgent need for novel methods that could help achieve the long-awaited progress in glioblastoma treatment. One of the potentially promising approaches is the targeting of non-coding RNAs (ncRNAs). These molecules are characterized by extremely high multifunctionality and often act as integrators by coordinating multiple key signaling pathways within the cell. Thus, the impact on ncRNAs has the potential to lead to a broader and stronger impact on cells, as opposed to the more focused action of inhibitors targeting specific proteins. In this review, we summarize the functions of long noncoding RNAs, circular RNAs, as well as microRNAs, PIWI-interacting RNAs, small nuclear and small nucleolar RNAs. We provide a classification of these transcripts and describe their role in various signaling pathways and physiological processes. We also provide examples of oncogenic and tumor suppressor ncRNAs belonging to each of these classes in the context of their involvement in the pathogenesis of gliomas and glioblastomas. In conclusion, we considered the potential use of ncRNAs as diagnostic markers and therapeutic targets for the treatment of glioblastoma.

## INTRODUCTION

Gliomas form a heterogeneous group of primary brain tumors, grade IV astrocytoma (also known as glioblastoma (GBM)) being the most aggressive amongst them [1]. Treatment of patients with GBM has remained almost unchanged over the past 20 years. First, maximal surgical resection of the tumor is performed, followed by a course of radiotherapy often supplemented with chemotherapy using temozolomide (TMZ), a DNA alkylating agent. However, despite this combination treatment, the mean survival rate of patients with GBM is extremely low compared to that for other cancer types. Thus, the 5-year survival rate of these patients is 4–5%, while the 2-year survival rate is approximately 26–33%.

Today, a mutation in the *IDH *gene and the level of* MGMT *promoter methylation are the key prognostic markers of gliomas widely used in clinical practice. The IDHR132H mutation detected in almost 50% of all glioma specimens alters the metabolism and causes histone hypermethylation; strangely enough, this significantly increases patients’ chances of survival [2]. The *MGMT *promoter methylation revealed in ~ 40% of all GBM specimens correlates with susceptibility to TMZ and is associated with a favorable outcome for patients receiving radiation therapy and chemotherapy [3]. Laboratory studies and an analysis of genome and transcriptome databases have allowed us to identify other survival-related markers and classify glioblastomas into phenotypic groups differing in terms of tumor aggressiveness and susceptibility to therapy [4]. However, none of these approaches has gained a foothold in clinical practice thus far.

The past decades have witnessed a vigorous search for novel drugs for the treatment of glioblastoma. In particular, low-molecular-weight compounds inhibiting receptor tyrosine kinases such as EGFR (dacomitinib; phase II trials) and PDGFR (sunitinib; phase II/ III trials), as well as epigenetic regulator proteins such as HDAC6 (panobinostat; phase II trials), are being studied. However, although similar drugs have proved highly effective in the treatment of various types of cancer, no encouraging results have been witnessed yet for glioblastoma [5, 6]. Along with low-molecular-compounds, a humanized monoclonal antibody against vascular endothelial growth factor A (VEGFA), known as bevacizumab, has been approved in a number of countries. However, it was shown later that bevacizumab, in combination with standard treatment, does not significantly increase a patient’s survival [7]. Injecting immune cells exhibiting direct antitumor activity is another promising method to treat GBM. Some immunotherapy variants are currently undergoing different phases of clinical trials [8], but none of them is actively used in clinical practice.

Various classes of non-coding RNAs (ncRNAs) that often play an extremely important role in the regulation of the vitality of tumor cells are a rather promising target for developing new methods for glioblastoma treatment. An evident challenge related to the design of these drugs is that compounds capable of specifically interacting with a target nucleic acid sequence need to be used. This significantly increases the minimal size of a drug molecule and impedes its penetration through the cell membrane. In this review, we have made an attempt to systematize the data on the non-coding RNAs involved in the glioma pathogenesis and discuss the therapeutic strategies related to them.

Over the past two decades, it has become increasingly clear that non-coding transcripts play a crucial role both in natural physiological processes and in the development of various diseases, including cancer [9]. It has been found that ncRNAs are also involved in the pathogenesis of malignant glial tumors. Many ncRNAs have pro-oncogenic properties. Their level in malignant tumor tissues is significantly higher than in normal brain tissues. In many cases, expression of the respective ncRNA correlates with disease stage and (or) tumor phenotype [10, 11]. The ncRNAs associated with pro-neural to mesenchymal transition, proliferation of tumor stem cells, as well as ncRNAs facilitating tumor adaptation to hypoxia, are known [11, 12, 13]. Furthermore, it has been reported that oncogenic ncRNAs can both be synthesized in tumor cells and migrate to other cells within exosomes and microvesicles, which may contribute to further disease progression [14]. Meanwhile, numerous ncRNAs functioning as tumor suppressors have been reported [15, 16, 17, 18]. Therefore, the information on the expression of numerous ncRNAs can theoretically be an important prognostic factor for patients. On the other hand, understanding the mechanism via which ncRNAs affect the key cellular processes can open up new prospects for the development of novel medications for the treatment of malignant glial tumors. In this review, we focus on long non-coding RNAs (lncRNAs), circular RNAs (circRNAs), microRNAs (miRNAs), PIWI-interacting RNAs (piRNAs), small nuclear RNAs (snRNAs), and small nucleolar RNAs (snoRNAs) in the context of their impact on the development of malignant glial tumors in humans (*Figure*). The roles played by transfer RNAs or ribosomal RNAs lie beyond the scope of our review; so, we will not discuss them.

**Fig. 1:**
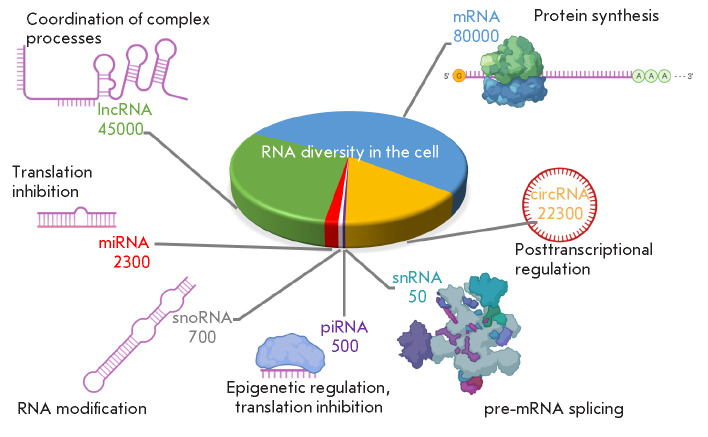
Functions of different types of ncRNAs in the cell. The diagram in the center and the numbers next to it indicate the approximate number of transcripts belonging to different classes of ncRNAs expressed in human cells [19, 20, 21, 22, 23, 24]. The inscriptions indicate the main function of the corresponding group of ncRNAs

## 1. LONG NON-CODING RNAS

**1.1. Biosynthesis, classification, localization, and functions of lncRNAs**

The group of lncRNAs includes nontranslated RNAs ≥ 200 nucleotides long. According to different estimates, 15,000 to 50,000 lncRNAs have been identified in humans [9, 25]. Most of these RNAs form with the involvement of RNA polymerase II; however, transcription of some lncRNAs can involve RNA polymerase III [26]. These RNAs are not translated for two reasons. First, their sequence usually does not contain open reading frames longer than 300 nucleotides. Second, these RNAs can contain various inactivating mutations that disable translation [27, 28]. As reported recently, some lncRNAs contain short open reading frames and can be translated to produce peptides whose function still needs to be elucidated in most cases [29]. Similar to mRNAs, lncRNAs can be capped and polyadenylated. Meanwhile, lncRNAs not carrying these modifications (e.g., lincROR) are also known [27]. According to the GenBank data, many lncRNAs (NEAT1, GAS5, and MALAT1) can undergo splicing, including alternative splicing, to produce several isoforms. Some lncRNAs (MALAT1 and GAS5) are widely expressed in most human tissues, while others (CRNDE and HOTAIR) are present only in certain types of tissues (the GenBank data). Furthermore, it is known that some lncRNAs (H19) are transcribed only during embryonic development, while their elevated level in the tissues of adult humans is indicative of pathology [30].

There are several criteria that are used for lncRNA classification: the position of the respective gene, the size, intracellular localization, and functions. The classification based on genomic localization of the lncRNA gene is provided below [9]. According to this classification, there are: (1) *intergenic *lncRNAs whose sequences do not overlap with those of protein-coding genes; (2) *antisense *lncRNAs that are transcribed in the direction opposite to the protein-coding genes and overlap with the gene sequences either partially or completely; (3) *bidirectional *(*or divergent*) lncRNAs whose transcription is initiated near the gene promoter and proceeds in the opposite direction; (4) *intronic* (sense and antisense) lncRNAs whose transcription is confined to gene introns; (5) *pseudogene-derived lncRNAs*, which are the transcripts of gene copies that have lost their coding potential due to inactivating mutations; (6) *telomeric and subtelomeric *lncRNAs that are transcribed from the telomeric chromosomal regions and contain telomeric sequences; (7) *centromeric *lncRNAs that are transcribed from centromeric regions and contain centromeric repeats; (8) *promoter-associated* lncRNAs; and (9) *enhancer-associated *lncRNAs that are expressed from these regulatory elements of the genome in both directions [9].

The lncRNAs can localize both inside the cell nucleus and in the cytoplasm [9]. Cytoplasmic lncRNAs can entrap regulatory miRNAs and various proteins, thus impeding their effects on the respective targets [31, 32]. The lncRNAs can ensure stability of other RNAs in the cytoplasm by binding to them [33]. Some lncRNAs act as precursors of regulatory miRNAs [34]. Nuclear lncRNAs can regulate gene expression by recruiting chromatin remodeling proteins and various activating or repressive complexes to gene promoters. Finally, due to their size, lncRNAs can act as scaffolds for the assembly of macromolecular protein complexes [35]. Furthermore, lncRNAs can stabilize chromosome loops by ensuring interaction between gene enhancers and promoters [36]. Some lncRNAs play a structure-forming role by being involved in the formation and maintenance of certain nuclear structures [37]. A number of lncRNAs have also been shown to play a crucial role in the occurrence of genomic imprinting and X chromosome inactivation [9].

The numerous lncRNAs that have been described can be viewed as prognostic markers for malignant glial tumors. Some of them have pro-oncogenic functions, while others act as tumor suppressors. However, existing data on many transcripts are very controversial. Some studies indicate that the same lncRNA can act as an oncogene for glioblastoma and as a tumor suppressor for other types of glioma that are less malignant. Thus, this finding is true for lincROR [38, 39]. We will focus on several lncRNAs that play different roles in the progression of GBM as an example. *Table 1* summarizes the remaining lncRNAs whose functions in glioblastoma cells have been studied.

**Table 1:** The role played by lncRNAs and circRNAs in the pathogenesis of malignant glial tumors

Name	Type of ncRNA	Role	Molecular mechanism of action	Reference
lncRNAs				
H19	Intergenic	Oncogene	Is a precursor of miR-675; acts as a ceRNA for microRNA Let-7.	[34, 40]
HOTAIR	Antisense	Oncogene	Recruits chromatin modeling complexes PRC2 and CoREST; acts as a ceRNA for many miRNAs (e.g., miR-326).	[35, 41]
CRNDE	Divergent	Oncogene	Recruits chromatin modeling complexes PRC2 and CoREST; acts as a ceRNA for many miRNAs (e.g., miR-186).	[42, 43]
XIST	Intergenic	Oncogene	Acts as a ceRNA for many miRNAs (e.g., miR-152).	[44]
NEAT1	Intergenic	Oncogene	Acts as a ceRNA for many miRNAs (e.g., miR-107); recruits EZH2 to promoters of the AXIN2, ICAT and GSK3B genes.	[45, 46]
PVT1	Intergenic	Oncogene	Acts as a ceRNA for many miRNAs (e.g., miR-128-3p); interacts with EZH2.	[47, 48]
CASC2	Divergent	Tumor suppressor	Acts as a ceRNA for miR-21.	[49]
GAS5	Divergent	Tumor suppressor	Acts as a ceRNA for miR-222.	[50]
PTENP1	Pseudogenic	lncRNA	Tumor suppressor Acts as a ceRNA for many miRNAs regulating PTEN expression.	[15, 31]
lincROR	Intergenic	Dual	Acts as a ceRNA for miR-145.	[51]
MEG3	Intergenic	Dual	Contributes to p53 stabilization; acts as a trap for miR-19a.	[52, 53]
NEAT2/MALAT1	Intergenic	Dual	Acts as a ceRNA for many miRNAs (e.g., miR-384).	[54]
HOTTIP	Antisense	Dual	Acts as a ceRNA for miR-101.	[55]
circRNAs				
circHIPK3	Exonic	Oncogene	Acts as a ceRNA for some miRNAs (e.g., miR-654).	[56]
circPVT1	Exonintronic	Oncogene	Acts as a ceRNA for miR-199a-5p.	[57]
circCFH	Exonic	Oncogene	Acts as a ceRNA for miR-149.	[58]
circTTBK2	Exonic	Oncogene	Acts as a ceRNA for some miRNAs (e.g., miR-761).	[59]
circSMARCA5	Exonic	Tumor suppressor	Interacts with splicing factor SRSF1, thus preventing the formation of oncogenic transcripts.	[60]
circFBXW7	Exonic	Tumor suppressor	Encodes the protein promoting ubiquitin-dependent degradation of c-Myc.	[16]
circSHPRH	Exonic	Tumor suppressor	Encodes the protein protecting SHPDH protein against ubiquitin-dependent degradation.	[17]
circPINT	Exonic	Tumor suppressor	Encodes the peptide increasing the affinity for the PAF1 complex to the target genes.	[18]
circITCH	Exonic	Tumor suppressor	Acts as a ceRNA for miR-214.	[61]

**1.2. Oncogenic lncRNAs**

NEAT1 (nuclear enriched abundant transcript 1, or nuclear paraspeckle assembly transcript 1) is an interesting example of oncogenic lncRNAs that has been well studied in glioblastomas. The intron-lacking* NEAT1 *gene resides on chromosome 11q13.1. A full-length 22,743-nucleotide-long non-polyadenylated transcript of NEAT1 and a 3735-nucleotide-long truncated polyadenylated lncRNA have been revealed (the GenBank data). NEAT1 is needed for the formation of paraspeckle nuclear condensates [37], ribonucleoprotein bodies sized 0.3–3 •µm surrounded by chromatin [62]. Pro-oncogenic protein SRSF1 is an important posttranscriptional regulator of NEAT1: it interacts with this lncRNA, thus enhancing its stability [63].

The NEAT1 content in glioblastomas is more than twofold higher than that in less aggressive types of gliomas. Furthermore, the level of this lncRNA in glioblastoma stem cells (CD133+) is twice higher than that in the less aggressive but better differentiated population of CD133- GBM cells [45]. Most often, NEAT1 exhibits its oncogenic effect in gliomas by binding to various miRNAs (e.g., miR-107) [45]. Moreover, NEAT1 recruits EZH2 to promoters of the *AXIN2, ICAT, *and* GSK3B *genes, thus facilitating H3K27 histone trimethylation and reducing the expression level of the aforementioned genes [46]. This example reveals a feature shared by all lncRNAs: they are able to activate different signaling pathways; these pathways eventually result in identical changes in the cellular phenotype and, thus, enhance each other’s action.

**1.3. Tumor-suppressive lncRNAs**

GAS5 (grow arrest-specific 5) is one of the lncRNAs that suppress glioblastoma development. The *GAS5* gene residing on chromosome 1q25.1 partially overlaps with the 5’ end of the *ZBTB37 *gene transcribed in the opposite direction. Fifteen isoforms of lncRNA GAS5 differing in terms of length and the number of exons have been reported. The full-length non-polyadenylated transcript (725 nucleotides long) consists of 13 exons. The shorter isoforms contain 9–2 exons (the GenBank data). GAS5 interacts with the DNA-binding domain of the receptors of steroid hormones (glucocorticoids, mineralcorticoids, androgens, and progesterone), thus preventing them from impacting the target genes [64].* In vitro *experiments have demonstrated that lncRNA GAS5 acts as a tumor suppressor in gliomas. Thus, X. Zhao *et al*. (2015) found that GAS5 inhibits the proliferation of U87 and U251 cells by binding to oncogenic miR-222 [50]. Furthermore, GAS5 overexpression increases the susceptibility of U87 cells to cisplatin [65]. Clinical trials also demonstrate that an increased GAS5 level correlates with a more favorable prognosis both in patients with glioblastoma and less malignant gliomas [66].

**1.4. The lncRNAs exhibiting dual effect on glioma cells**

Along with lncRNAs that play either an oncogenic or a tumor-suppressor role, there are several lncRNAs whose functions depend on the context. NEAT2/ MALAT1 (metastasis associated lung adenocarcinoma transcript 1) is one of such lncRNAs. The *MALAT1* gene residing on chromosome 11q13 is expressed in various human tissues, including the brain. Three variants of lncRNA MALAT1 having a similar size (~ 8000 nucleotides) have been described; they are produced by splicing and differ in terms of the number of exons (the GenBank data). During MALAT1 processing, a small fragment is cleaved from the 3’ end of the primary transcript and is transferred to the cytoplasm. The mature lncRNA MALAT1, ~ 7,000 nucleotides long, predominantly remains inside the nucleus and localizes in nuclear speckles [67]. MALAT1 does not contain poly(A) sequences; however, it is rather stable, since a special triplex structure forms at its 3’ end. MALAT1 is associated with the splicing factors SRSF1, SRSF2, and SRSF3, and thus involved in mRNA processing. In addition, MALAT1 regulates gene expression at a transcriptional level. Thus, this lncRNA can bind to the nonmethylated protein Pc2 (polycomb 2 protein) to facilitate its interaction with the E2F transcription factor and transcriptional coactivators [67]. Meanwhile, the oncogenic role of MALAT1 in cancer is mainly related to its ability to affect the level of certain miRNAs (including miR-384) [54]. The meta-analysis conducted by Q. Zhou *et al*. (2018) demonstrated that an increased MALAT1 level correlates with an unfavorable prognosis in patients with glioma [68]. *In vitro *experiments have demonstrated that suppression of MALAT1 expression reduces cell resistance to temozolomide, as well as cell proliferation, migration, and invasion, and stimulates apoptosis [69]. Contrariwise, Y. Han* et al*. revealed that the MALAT1 level in gliomas is 1.5-fold lower than that in a normal brain. Furthermore, overexpression of MALAT1 reduces the proliferation of U87 and U251 cells [70]. It was also found that MALAT1 forms a complex with the RNA-binding protein HuR and ensures its recruitment to exon 2 of the *CD133 *gene, the key marker of glioblastoma stem cells. As a result, *CD133 *expression is suppressed at the transcriptional level [71]. Therefore, MALAT1 is involved in the fine tuning of the phenotype of glioblastoma cells while changes in the level of this RNA (both the increased and decreased levels) result in an unfavorable effect on cells.

## 2. CIRCULAR RNAs

**2.1. General characteristics, biosynthesis, classification, and functions**

circRNAs include transcripts whose 5’ and 3’ ends are linked by a phosphodiester bond yielding a circular structure. Inverted repeats contained in the precursors contribute to the formation of circRNAs [72, 73]. The circRNAs formed from RNA precursors via the so-called reverse splicing. Whereas the 5’-terminal donor site is bound to the 3’-terminal acceptor site in the case of canonical splicing, during reverse splicing, the 3’-donor site interacts with the 5’-acceptor site, thus producing a covalently closed circular transcript. According to some reports, reverse splicing (as well as the conventional forward one) occurs via the canonical spliceosome assembly pathway [73]. In a number of cases, both linear and circular RNAs can be transcribed from the same sequence [47, 57]. Depending on their origin and structure, there are: (1) *exonic circRNAs (ecircRNAs)*, (2) *exon*–*intronic circRNAs *(*eIcircRNAs*), (3) *intronic circRNAs *(*icircRNAs*), and (4) *intergenic circRNAs *(*igcircRNAs*). In the first case, circRNAs are formed from the mRNAs of protein-coding genes. As a result, this RNA can have the same exon composition as mRNA, but the 5’ end of exon 1 is connected to the 3’ end of the last exon in circRNAs. In the case of eicircRNAs, the circular transcripts contain some of the intronic sequences of RNA precursors. icircRNAs and igcircRNAs are formed upon transcription of the intronic and intergenic sequences, respectively [72]. Circular RNAs are neither polyadenylated nor capped. They are more stable than linear lncRNAs, and thus more promising diagnostic markers and therapeutics [72]. Importantly, transcription of linear and circular RNAs of the same gene can occur independently of each other as it was demonstrated for lncRNA PVT1 and circPVT1 [47, 57].

In a manner similar to lncRNAs, circRNAs can interact with other RNAs, DNAs, and proteins, as well as perform various functions in the cell. Many circRNAs contain microRNA binding sites and act as a “sponge” by adsorbing these molecules [56, 57, 58, 59, 61]. Circular transcripts can also compete with the mRNAs of protein- coding genes for splicing factors, thus reducing the efficiency of mRNA processing. A number of circRNAs act as adaptors and recruit various proteins, thus ensuring their interaction with each other. Furthermore, circRNAs can reside on gene promoters and regulate their transcription [73]. Although circRNAs are not capped, some of them contain short reading frames and are translated to produce small proteins and peptides [16, 17, 18]. The nucleotide sequences of these circRNAs contain the specific IRES elements required for the interaction with ribosomes and translation initiation factors [73].

It was not until recently that circRNAs were found to be involved in the pathogenesis of malignant glial tumors. Nevertheless, there already are several publications that have detected circular transcripts differentially expressed in patients with glioma and glioblastoma. These transcripts are now being actively studied, and many of them can be regarded as potential diagnostic markers. Some circRNAs playing a pro-oncogenic or tumor-suppressor role in the pathogenesis of malignant glial tumors will be listed below. *Table 1* provides a more detailed list of circRNAs with known functions.

**2.2. Pro-oncogenic circRNAs**

One of the pro-oncogenic circRNAs is circHIPK3. The* HIPK3 *(homeodomain interacting protein kinase 3) gene resides on chromosome 11p13 (the GenBank data). Several circRNAs generated by noncanonical splicing of the primary *HIPK*3 linear transcript are known. The 1099-nucleotide-long circular transcript involving only the *HIPK3 *exon 2 is most abundant in human tissues (the CircBase data). This transcript increases cell proliferation and acts as a trap for several miRNAs. P. Jin* et al*. (2018) showed that the circHIPK3 level in gliomas is 1.5- to 5-fold higher than that in the normal brain tissue of the same patients. Furthermore, the increased circHIPK3 level reduces the mean survival time in patients almost twofold [56]. Suppression of this circRNA in *in vitro *experiments reduces proliferation of U87 and U251 cells. It was found that circHIPK3 acts as a “sponge” for miR-654, which in turn regulates the level of pro-oncogenic protein IGF2BP3 [56].

**2.3. Tumor-suppressive circRNAs**

circSMARCA5 is an example of tumor-suppressor circRNA. The *SMARCA5 *protein-coding gene resides on chromosome 4 (4q31.21). The 269-nucleotide-long circSMARCA5 includes exons 15 and 16 (according to the CircBase data). This circRNA is highly transcribed in the human brain and plays an oncoprotective role. A reduced SMARCA5 level was shown to correlate with an unfavorable prognosis in patients with glioblastoma [60]. Overexpression of SMARCA5 contributes to reduced migration of U87MG cells. Circular RNA SMARCA5 contains binding sites for the splicing factor, which plays a pro-oncogenic role in many cancers, including glioblastomas. By interacting with SRSF1, SMARCA5 prevents its involvement in alternative splicing and the generation of oncogenic transcripts. In particular, this circRNA reduces the ratio between the oncogenic and anti-oncogenic VEGF-A isoforms [60].

## 3. SMALL NON-CODING RNAs

Small non-coding RNAs (sncRNAs) are small molecules 18–200 nucleotides long. Several types of sncRNAs have been identified thus far, namely, tRNAs, miRNAs, small interfering RNAs (siRNAs), small nuclear RNAs (snRNAs), small nucleolar RNAs (snoRNAs), telomerase RNA components (TERC), PIWI-interacting RNAs (piRNAs), small enhancer RNAs (seRNAs), and Y RNAs [74]. This list still continues to expand. By cooperating with other intracellular molecules, sncRNAs are involved in regulation of gene expression at all levels: the cotranscriptional, posttranscriptional, translational, and epigenetic ones. An improper amount and functions of sncRNAs alter the intracellular processes and trigger various diseases: not only cancer, but also neurodegenerative and cardiovascular diseases, etc. [75]. There are several reasons why the level of sncRNAs synthesized by the cell is altered. First, this occurs due to mutations in the genes encoding sncRNAs per se [76]. The second reason is the mutations and disrupted functions of the enzymes responsible for sncRNA biogenesis (e.g., Dicer and Drosha for miRNAs) [77]. The epigenetic, transcriptional, or posttranscriptional control over expression of both sncRNAs and enzymes processing them can also be disrupted [77]. In this section, we will focus on the types of sncRNAs involved in the pathogenesis of malignant glial tumors. *Table 2* provides brief characteristics of these sncRNAs.

**Table 2:** The key characteristics of the sncRNAs involved in glioblastoma pathogenesis

Parameter	miRNAs	piRNAs	snoRNAs	snRNAs
Length	~ 22 nucleotides	~ 24–30 nucleotides	~ 60–300 nucleotides	~ 80–350 nucleotides (on average, ~ 150 nucleotides)
Genomic localization	In the intronic regions of protein-coding genes, sometimes in exons	In PIWI clusters	In introns of protein-coding genes and polycistronic snoRNA clusters	In snRNA genes
Precursors	Double-stranded hairpin	RNA	Single-stranded	RNA Single-stranded RNA Single-stranded RNA
RNA polymerase performing transcription	RNA polymerase II	RNA polymerase II	RNA polymerase II	RNA polymerase II; for U6, RNA polymerase III
Mechanism of processing	Double-stage cleavage by Drosha and Dicer proteins	5’- and 3’-exonuclease-assisted truncation, followed by cleavage by Zucchini protein	Splicing of pre-mRNA,opening of the lariat structure, followed by its 5’- and 3’-exonuclease-assisted truncation	Capping and modification of the 3’-end of the molecule
Classes of RNAbinding proteins	Argonaute	PIWI	5.5 K, NOP56, NOP58, and firillarin	Spliceosomal proteins
Functions	Regulation of expression of protein-coding genes	Transposon silencing	Posttranscriptional modifications of the other types of cellular RNAs	pre-mRNA splicing

**3.1. microRNAs**

microRNAs (miRNAs) are short RNAs (~ 22 nucleotides long) involved in posttranscriptional regulation of gene expression. The sequences encoding miRNAs in most cases reside inside introns, although exonic miRNAs are sometimes found. Transcription of miRNAs is performed by RNA polymerase II, which also transcribes the host gene [78]. After the multi-stage processing that has been described in detail in many reviews [78], miRNAs within the RNA-induced silencing complex (RISC) is involved in recognition of target gene mRNAs. The crucial criterion for choosing the target mRNA is the presence of a domain complementary to the so-called *seed sequence *of a miRNA, which is a region consisting of six nucleotides (nucleotides 2 through nucleotide 7) at the 5’ end of a miRNA molecule [79]. These complementary domains are most typically found in the 3’-untranslated regions of mRNA (i.e., outside its protein-coding region). The complementarity (either complete or partial) ensures binding between the target mRNA and the RISC, which either causes mRNA degradation or represses its translation. In the former case, GW182 protein ensures the removal of poly(A) tail or 5’ cap from the mRNA molecule [80] to give rise to a non-functional product that is degraded by 5′-3′ exoribonuclease 1 (XRN1) [79]. There is currently no consensus regarding translational repression, but most studies indicate that the RISC causes dissociation of translation initiation factors eIF4AI and eIF4AII from the mRNA target, thus inhibiting mRNA scanning by the ribosome and formation of the translation initiation complex eIF4F [81]. Both the aforementioned gene silencing mechanisms are interrelated; however, according to the ribosome profiling data, 66–0% of gene silencing is caused by mRNA degradation [82]. The available estimates suggest that miRNAs are involved in expression regulation of approximately 30% of human genes [83]. The impact of a single miRNA on gene expression is usually appreciably weak. Therefore, miRNAs typically form large-scale networks of intracellular molecular interactions, thus exhibiting a synergistic effect. We would like to thoroughly describe several miRNAs playing different roles in progression of GBM as an example (*Table 3* lists miRNAs whose functions have been studied in glioblastoma cells).

**Table 3:** sncRNAs associated with glioblastoma development

miRNA	Role	Target genes	Function	Reference
miRNAs				
let-7	Tumor suppressor	NRAS, KRAS, CCND1	Reduces proliferation and invasion; increases apoptosis and susceptibility to cisplatin	[84]
miR-7	Tumor suppressor	EGFR, FAK, PI3K, RAF1	Reduces invasion and migration	[84]
miR-17	Tumor suppressor	PTEN, MDM2, CCND1, AKT1	Reduces cell migration and viability	[84, 85]
miR-21	Oncogene	ANP32A, SMARCA4, RECK, TIMP3, IGFBP3	Enhances proliferation, invasion, and chemoresistance	[86, 87]
miR-24	Oncogene	ST7L, SOX7	Enhances proliferation and migration	[88]
miR-221/222	Oncogene	PTEN, PUMA, MGMT	Enhances proliferation, invasion, and treatment resistance	[84]
miR-326	Tumor suppressor	NOTCH1, NOTCH_2_	Reduces cell viability	[84, 89]
miR-451	Tumor suppressor	CAB39, LKB1, AMPK, PI3K, AKT	Inhibits proliferation	[84, 90]
piRNAs				
piR-30188	Tumor suppressor	lncRNAs OIP5-AS1	Reduces proliferation, migration, and invasion of glioma cells and stimulates apoptosis	[91]
piR-8041	Tumor suppressor	MAP3K76, RASSF1	Induces cell cycle arrest and reduces proliferation	[92]
piRDQ593109	Tumor suppressor	Causes degradation of miR-330-5p	Loosens the tight intercellular junctions	[93]
piR-598	Tumor suppressor	BAX, GOS2, JUN	Enhances apoptosis and reduces proliferation	[94]
snoRNAs				
SNORD44	Tumor suppressor	CASP3, CASP8, CASP9	Induces apoptosis, reduces proliferation and invasiveness	[95]
SNORD47	Tumor suppressor	CCNB1, CDK1, CDC25C, CTNNB1, CDH2, VIM, MMP2, MMP9, CDH1	Inhibits proliferation and increases patients’ survival	[96]
SNORD76	Tumor suppressor	CCNA1, CCNB1	Inhibits growth and proliferation of glioma cells	[97]
snRNAs				
U1	Oncogene	The mutation in U1 inactivates PTCH1 and activates GLI2 and CCND2	Upregulates oncogene expression and inactivates tumor suppressor genes	[98]

*3.1.1. Oncogenic microRNAs. *The findings reported in numerous studies describing the role played by oncogenic miRNAs in the pathogenesis of gliomas have been published [94, 95]. Tumor suppressor genes usually act as targets for these miRNAs, while the disruption of miRNA expression causes uncontrolled cell proliferation, enhances cell migration and invasion, induces angiogenesis and blocks apoptosis. miR-21 is one of the best-studied oncogenic miRNAs; its level is elevated in many cancers and correlates with disease grade in gliomas [10]. This miRNA regulates numerous intracellular processes promoting glioma development [86]. The miR-21 targets include the genes promoting apoptosis (*PDCD4 *and *LRRFIP1*) [99, 100], as well as the tumor suppressor genes inhibiting invasion (*RECK *and *TIMP3*) [101] and proliferation (*IGFBP3*) [87]. Furthermore, miR-21 can affect microglial behavior, thus ensuring favorable conditions for tumor growth. miR-21 was detected in vesicles secreted by glioma cells [14]. Having entered microglia, the vesicles reduced expression of the target genes of miR-21 (*Bmpr2*, *Btg2*, *Kbtbd2*, *Pdcd4*, *Pten*, and *Rhob*). Some of these genes are involved in cell proliferation and differentiation. Therefore, their inhibition by vesicular miR-21 enhanced microglial proliferation, which may significantly affect the formation of tumor microenvironment and promote its progression as suggested in ref. [14].

Interestingly, more and more data on the important role played by exogenous miRNA molecules (the ones coming from neighboring cells) are being collected. Thus, oncogenic miRNAs can migrate between glioma cells and their microenvironment (astrocytes, oligodendrocytes, endothelial cells, and microglia/ macrophages), thus being involved in intercellular communication, which contributes to tumor progression [14]. Co-culturing astrocytes with glioma cells increases the levels of nine miRNAs (miR-4519, miR-5096, miR-3178, etc.) in astrocytes; two miRNAs (miR-5096 and miR-4519) directly migrate to astrocytes from glioma cells through gap junctions [102]. The miRNA transfer in the opposite direction has also been reported: miR-19a is transferred from astrocytes to tumor cells by vesicles and inhibits PTEN activity in tumor cells, thus causing metastatic growth. Furthermore, the exosomes secreted by hypoxic glioma cells induce polarization of M2 macrophages and exhibit an immunosuppressive effect, thus promoting glioma proliferation, migration, and invasion *in vitro* and *in vivo*. This effect is attributed to the presence of miR-1246 in exosomes [103].

*3.1.2. Tumor-suppressive miRNAs. *A large number of tumor-suppressive microRNAs are known [84]. Thus, miR-7 inhibits signal transduction through the EGF receptor involved in the Akt protein kinase signaling pathway. However, miR-7 expression is suppressed (its level is reduced more than sixfold compared to the normal tissues) in glioblastoma, so the Akt signaling pathway is permanently activated and the viability and proliferation of tumor cells is increased [104]. It has also been demonstrated that exogenous administration of proapoptotic miR-218 suppresses expression of cyclin-dependent kinase 6 (CDK6), reduces proliferation, and causes apoptotic death of glioma cells [105]. Another target of miR-218 is EGFR-coamplified and overexpressed protein (ECOP), which regulates the transcriptional activity of NF-κB. Overexpression of miR-218 in glioma cells leads to a curb of the activity of NF-κB by ECOP by causing apoptosis and slowing down proliferation [106].

**3.2. PIWI-interacting RNAs (piRNAs)**

PIWI-interacting RNAs (piRNAs) are the non-coding RNAs approximately 24–35 nucleotides long which were initially detected in the Drosophila gonads. These RNAs have got their name because they bind to PIWI (P-element-induced wimpy testis) proteins [107, 108]. The so-called piRNA clusters that mainly reside in the intergenic or non-coding domains are the sources of piRNAs in the genome [109]. There are two mechanisms for piRNA formation in the cell: (1) via the primary processing pathway and (2) via the ping–pong mechanism resulting in amplification of secondary piRNAs. These mechanisms have been thoroughly described in reviews [110, 111]. It has been demonstrated that piRNAs are involved in the pathogenesis of various diseases, including malignant neoplasms [112, 113]. According to the profiling data, approximately 350 piRNAs are expressed in normal brain tissues and GBM, some piRNAs being typical of GBM only [92].

*3.2.1. Oncogenic piRNAs. *Because piRNAs have recently been studied in various types of malignant tumors, only a few publications focusing on piRNAs in gliomas are available, and there are no publications that would disclose the oncogenic role played by piRNAs in the development of glial tumors.

*3.2.2. Tumor suppressor piRNAs. *Database analysis has revealed that single-nucleotide polymorphisms in the piR-2799, piR-18913, piR-598, piR-11714, and piR-3266 genes are associated with the increased risk of glioma development; the piR-598 variants correlate with a risk level stronger than other variants do. The transcriptome profiling of cells transfected with wild-type piR-598 indicates that this piRNA affects expression of 518 genes involved in glioma cell death/survival. The presence of piR-598 reduced expression of most of the detected genes (71.2%). The gene encoding the oncogenic transcription factor Jun is one of the genes whose expression was significantly decreased. Simultaneously, piR-598 increases the level of BAX and GOS2 pro-apoptotic proteins. Studies focused on the effect of piR-598 on *in vitro *growth of glioma cells demonstrated that overexpression of wild-type piR-598 reduces cell proliferation and colony formation; contrariwise, overexpression of the mutant piR-598 increases them, which is consistent with the transcriptome analysis data [94]. However, the exact mechanisms underlying these processes have not been elucidated yet and need to be studied further. Other tumor suppressor piRNAs are listed in *Table 3*.

**3.3. Small nucleolar RNAs**

Small nucleolar RNAs (snoRNAs) are localized in the nucleolus and are 60–300 nucleotides long. Human snoRNAs reside in the intronic domains of the genes encoding proteins or lncRNAs and are cut out from them during splicing [114]. snoRNAs have several functions, their involvement in processing and maturation of other types of cellular RNAs being the best-known function. Therefore, three classes of snoRNAs have been differentiated: C/D box snoRNAs (involved in 2′-O-methylation of rRNAs), H/ACA box snoRNAs (involved in pseudouridination of RNA nucleotides), and small Cajal body-specific RNAs (cbsRNAs belonging to the class of box C/D–H/ACA RNAs and involved in 2′-O-methylation and pseudouridination of spliceosomal U1, U2, U4, and U5 snRNAs) [114]. snoRNAs were reported to act both as tumor suppressors and oncogenes. They are known to be involved in proliferation, apoptosis, metastasizing, and the development of drug resistance by tumor cells, while the mechanisms of action of these RNAs differ [115].

*3.3.1. Oncogenic snoRNAs. *No oncogenic snoRNAs involved in glioma development have been reported thus far.

*3.3.2. Tumor suppressor snoRNAs. *SNORD47 is one of the tumor suppressor snoRNAs whose level in gliomas is twice lower compared to that in normal brain tissues. A comparison of gliomas of different grades showed that most grade III–IV gliomas have a significantly reduced SNORD47 level (revealed in 71.4% of the analyzed specimens). Therefore, the survival of patients with a higher SNORD47 expression in glioma tissues is better compared to that in patients with lower SNORD47 expression. Overexpression of SNORD47 results in the inhibition of cell proliferation by inducing cell cycle arrest in the G2 phase. This possibly takes place due to a downregulated expression of such important cell cycle regulators as cyclin B1, CDK1 and CDC25C, β-catenin, and phospho-β-catenin. The levels of N-cadherin, vimentin, and metalloproteinases 2 and 9 decrease simultaneously, and the level of E-cadherin increases, thus indicating that SNORD47 prevents the pro-neural to mesenchymal transition of glioma cells. Furthermore, SNORD47 overexpression increases the susceptibility of glioma cells to temozolomide [96]. SNORD44 is another tumor-suppressor snoRNA. Its level and the level of the transcript of its host gene, lncRNA GAS5, in glioma cells are 2–3 times lower than those in a healthy brain. The levels of caspase 3, caspase 8, and caspase 9 are elevated upon SNORD44 overexpression, thus causing apoptosis. Moreover, cells transfected with SNORD44 are characterized by a noticeably lower proliferation and invasiveness [116]. However, the exact molecular mechanisms of these processes remain unknown. Other examples of tumor suppressor snoRNAs are listed in *Table 3*.

**3.4. Small nuclear RNAs**

Small nuclear RNAs (snRNAs) consist of approximately 150 nucleotides. The U6 and U6ATAC snRNAs are synthesized by RNA polymerase III, while the remaining ones are by RNA polymerase II [117, 118]. During maturation, snRNAs undergo numerous processing and folding stages and bind to various proteins to form functional snRNPs. Mature snRNPs are imported back to the nucleus and travel to Cajal bodies to perform their functions. The snRNA biogenesis is discussed in more detail in review [119].

The key function of snRNAs is participation in pre-mRNA processing. snRNAs are the spliceosome components: U1, U2, U4, U5, and U6 are the components of the major spliceosome, while U5, U11, U12, U4ATAC, and U6ATAC are the components of the minor one. U7 and U8 have extra-spliceosomal functions: U7 is involved in the processing of histone pre-mRNA [120], while U8 is needed for rRNA maturation [121]. The involvement of snRNAs in splicing was thoroughly described earlier [122, 123]. The normal functioning of all components of the splicing machinery is critical for many biological processes: so, it is not surprising that splicing disruption is observed in multiple diseases, including glioblastoma [124].

*3.4.1. Oncogenic snRNAs. *Mutations in snRNAs are detected in various types of cancer [25], including brain tumors. Thus, mutations in the third nucleotide within the binding domain of the 5′-splice site in U1 were detected in medulloblastoma cells. Alternative splicing results in inactivation of tumor-suppressor genes (*PTCH1*) and activation of oncogenes (*GLI2 *and* CCND2*) in medulloblastoma cells with mutant U1 sn- RNA [98]. Vesicles secreted by apoptotic glioblastoma cells were also shown to contain spliceosome components, including U2, U4, and U6 snRNAs. The exogenous spliceosome components alter pre-mRNA splicing in recipient cells, making the tumor more aggressive and treatment-resistant [126].

*3.4.2. Tumor suppressor snRNAs. *Data on the tumor suppressor functions of protein splicing factors has been obtained, but nothing is known yet about the tumor suppressor function of snRNAs in gliomas.

## 4. APPLICATION OF NON-CODING RNAs IN TREATMENT AND DIAGNOSIS OF BRAIN TUMORS

Protein molecules have long been viewed as potential targets for antitumor therapy and markers of malignant neoplasms. However, the role played by the non-coding part of the genome in cell functioning identified over the past decades has offered new insight into cancer development mechanisms. The number of reports on ncRNAs that can be used either as antitumor therapy targets or as prognostic markers increases year by year [127, 128]. Furthermore, ncRNA-based drugs effective in the treatment of some diseases have already been designed [129].

Thus, many sncRNAs are found in the body fluids (blood plasma and serum or cerebrospinal fluid) of patients with gliomas. sncRNAs usually reside in exosomes, so they are protected against degradation and can pass through the blood–brain barrier (BBB) [130]. For this reason, sncRNAs can be used as good biomarkers in non-invasive diagnostics. For example, the miR-221 level in glioma tissue specimens and the blood plasma of patients is elevated 2–11 times. Its level increases with tumor grade. Therefore, miR-221 can be viewed as a potential diagnostic marker of glial tumors [131]. Similar results have also been obtained for miR-21 [132, 133]. Along with miRNAs, other types of sncRNAs can also act as potential biomarkers. Thus, the miR-320/miR-574-3p/RNU6-1 combination or RNU6-1 isolated from serum exosomes is specific to patients with glioblastoma [134].

New cancer treatment strategies based on the use of antisense oligonucleotides with various RNAs (including lncRNAs) acting as targets are currently being developed [135, 136]. However, the BBB significantly reduces the bioavailability of such therapeutics in patients with brain tumors of glial origin. It is more promising to use low-molecular-weight compounds showing highly specific binding to certain sequences (or certain structural motifs) of lncRNAs for GBM treatment. Thus, the compounds AC1NOD4Q and AC1Q3QWB bind to the region residing in the 5’-terminal domain of the oncogenic lncRNA HOTAIR and disrupt its interaction with EZH2, the catalytic subunit of the chromatin remodeling complex. These compounds significantly reduce the migration and invasion of glioma cells, as well as suppress their pro-neural to mesenchymal transition [137, 138, 139]. Compounds interacting with the specific triplex structure localized on the 3’ end of lncRNA MALAT1 have also been identified. These low-molecular- weight compounds can reduce the MALAT1 level and slow tumor growth in a mouse model of breast cancer [140].

RNP complexes containing snRNAs are a promising therapeutic target. It has been demonstrated that activity of U2-snRNP is needed for glioblastoma stem cells to survive and pass through the mitotic phase. Pladienolide B, a macrolide inhibiting activity of the SF3b subcomplex, disturbs the normal interaction between U2 snRNA and pre-mRNA, thus disrupting splicing and causing tumor cell death [141]. Two other antitumor agents, spliceostatin A and E7107, have the same effect [142, 143]. These agents disrupt mRNA splicing in such cell-cycle regulators as cyclin A2 and Aurora A kinase [144] by inhibiting the proliferation of tumor cells [145]. Furthermore, disrupted splicing results in the emergence of aberrant proteins, which may also cause tumor cell death [142]. Novel drugs aimed at splicing inhibition are being actively developed. For example, agent H3B-8800 is currently undergoing phase I clinical trials and is expected to become the first antitumor splicing inhibitor [146].

piRNA can become another potential target for the development of new therapy protocols. Drug delivery poses a significant problem relative to the treatment of brain tumors. Because of the blood–brain barrier, most agents cannot be delivered to the tumor at sufficient concentrations. However, S. Shen *et al*. have recently demonstrated that the penetrability of the blood–brain barrier can be increased by inhibiting the PIWIL1/piR-DQ593109 complex in the endothelial cells lining tumor blood vessels in gliomas [147]. This complex plays a crucial role in the degradation of oncogenic lncRNA MEG3, which in turn regulates the formation of tight intercellular junctions. PIWIL1/piR-DQ593109 knockdown increases the MEG3 level, eventually enhancing the permeability of the capillaries supplying the tumor with blood. This approach can be used to elaborate novel glioma treatment regimens.

## CONCLUSIONS

The research conducted over the past decades has made it clear that the roles of RNAs are not confined to protein coding. Due to their complex architecture and an ability to get involved in highly specific complementary interactions with a number of various molecules, ncRNAs can act as master regulators of crucial intercellular processes. Furthermore, ncRNAs were found to play a key role in intercellular interplay. It is therefore not surprising that more and more scholars are focusing their attention on the role played by these molecules in cancer, as well as the prospects of using them as a target for the development of novel antitumor agents. Unfortunately, it is much more challenging today to design a drug that would inhibit a specific ncRNA than to develop novel low-molecular-weight protein inhibitors. However, for aggressive cancer types such as glioblastoma, these very approaches can yield the long-awaited progress in patient treatment.

## Abbreviations

BBB: blood-brain barrier
ceRNA: competing endogenous RNA
circRNA: circular RNA
GBM: glioblastoma
lncRNA: long non-coding RNA
miRNA: microRNA
nc: nucleotide
ncRNA: non-coding RNA
piRNA: PIWI-interacting RNA
sncRNA: small non-coding RNA
snRNA: small nuclear RNA
snoRNA: small nucleolar RNA
TMZ: temozolomide
